# Editorial: Frontiers in diagnostic and therapeutic approaches in diabetic sensorimotor neuropathy, volume II

**DOI:** 10.3389/fendo.2026.2009636

**Published:** 2026-10-20

**Authors:** Zoltán Kender, Péter Kempler

**Affiliations:** 1 University of Heidelberg, Heidelberg, Germany; 2 Petz Aladar University Teaching Hospital, Department of Endocrinology, Diabetology and Metabolism, Győr, Hungary; 3 Semmelweis University, Department of Medicine and Oncology, Budapest, Hungary

**Keywords:** diabetic sensorimotor polyneuropathy (DSPN), neuroimaging, neuropathic pain, precision medicine, sensory phenotyping

For many decades, retinopathy was the most frequently investigated microvascular complication among diabetic patients. Nephropathy is associated with the worst prognosis; however, neuropathy is associated with high mortality and with poor quality of life as well.

Neuropathy predicts early renal impairment in diabetes mellitus (1) – the almost forgotten historical evidence was first published (2) by Göran Sundkvist in 1993 and has been recently confirmed in a 15 year long prospective study (3). It is not surprising that after two years, Front Endocrinol (Lausanne) has once again chosen neuropathy as a topic for a special issue.

Neuropathy is an early complication of diabetes. It is well known that it might be present among patients with prediabetes. Assessing three groups, prediabetes (HbA1c: 5,70-6,49%) and increased risk of prediabetes (HbA1c <5,7%, FINDRISC score ≥12) emerged as potential determinants of CAN independently of age, compared to controls (HbA1c <5,7%, FINDRISC <12), OR~8,4 and OR~7,0, respectively (Bekeffy et al.). These data suggest that CAN may be present even in individuals at risk of prediabetes. Moreover, neural impairment in prediabetes and increased prediabetes risk may be more closely linked to broader metabolic risk factors beyond short-term glycaemic variability.

The close relationship between autonomic and somatic nerve involvement is further supported by prospective data from Tsilingiris et al. Among individuals with diabetes who were free of DSPN at baseline, established CAN independently predicted incident DSPN across three complementary definitions during a mean follow-up of three years. Reduced short-term HRV also identified individuals at increased risk and may therefore provide a practical marker for neuropathy risk stratification. In another study focusing on patients with type 2 diabetes and diabetic kidney disease, Felicio et al. reported a higher prevalence of CAN in advanced kidney disease and an association between vitamin D deficiency and impaired HRV indices. Although the cross-sectional design precludes causal inference, these findings emphasize the clinically relevant intersection of autonomic dysfunction, renal disease and metabolic factors.

Nonetheless, patients’ age seems to have a significant effect on the severity of sensory neuropathy in type 2 diabetes mellitus. In a study performed in a well-defined metropolitan area, 442 patients were assessed (mean age, 65,5 ± 11,2 years, mean diabetes duration 9,6 ± 8,9 years). Pressure perception was evaluated with a 10-g Semmes-Weinstein monofilament, a calibrated 128 Hz tuning fork was used for the detection of vibration sensing (Kiss et al.). Additionally, patients were asked three standardized questions: 1. “Have you ever had a non-healing ulcer on any of your limbs?” 2. “Do you experience persistent pain in any lower limb at rest lasting more than 5 minutes?” 3. “Do you feel discomfort in any lower limb at rest lasting more than 5 minutes?” Answers were recorded as “yes” or “no”. These findings suggest that in an aging population with type 2 diabetes, peripheral nerve damage identified by standard screening tools is more strongly associated with age, rather than with diabetes duration. Neuropathy symptoms were more frequently reported, regardless of the diagnostic tools used. This way, the self-developed simple questionnaire seems to be useful in everyday clinical practice (Kiss et al.). Data indicates that the two screening methods often identify different patients in routine outpatient practice. Abnormal results were more common with the tuning fork; this device is more widely used in Europe.

Some highly interesting case reports were published in this special issue as well. The role of dentistry is overall underevaluated, almost neglected in diabetology and this is even more valid for neuropathy. Early hyperaesthesia and late hypoaesthesia, both as manifestations of sensory neuropathy have been reported (Liptak et al.). A 14-year-old girl with poorly controlled type 1 diabetes for 10 years had gingivitis, angular cheilitis, severe plaque accumulation due to poor oral hygiene, generalized carious lesions and pericoronaritis at tooth 47. She had amplified pain reactions to dental procedures despite adequate local anaesthesia. Lower sensory thresholds reflecting generalized sensory hyperexcitability were documented during quantitative sensory testing (Liptak et al.).

In the case of a 55 year old man with long term poor glycaemic control, clinical examination revealed severe generalized periodontitis characterized by gingival recession, attachment loss and tooth mobility. Multiple carious lesions were also recorded. However, little or no pain was reported by the patient, being incongruent with the extent of pathology. He described diminished oral sensitivity during dental interventions and tended to underestimate the severity of his oral condition, which contributed to delays in seeking treatment. Severe, partially maximal hypoaesthesia was detected during quantitative sensory testing. Both patients illustrate the importance of glycaemic control, the need for interdisciplinary cooperation and individualised diagnostic and therapeutic strategies in dentistry (Liptak et al.).

A 22-year-old female patient with episodes of hypoglycaemia had numbness, tingling and burning pain of the limbs (Korei et al.). Multiple radiculopathies, sensorimotor polyneuropathy and later a contrast-enhancing tumour at the pancreatic body-trail transition were diagnosed. After laparoscopic distal pancreatectomy prompt resolution of hypoglycaemia and alleviation of neuropathy-symptoms was observed. Insulinoma should be considered as a rare origin of neuropathy and early diagnosis is essential.

The reviews included in this Research Topic broaden this perspective considerably. The outstanding review by Okdahl and Brock reframes diabetic neuropathy as a disorder of the entire neuroaxis rather than an exclusively peripheral complication. It integrates somatic and autonomic neuropathy with diabetes-related alterations of the spinal cord, brainstem and brain, and highlights bidirectional interactions between peripheral nerve injury, central neuroplasticity and altered pain modulation. This holistic concept is particularly important for understanding the frequent dissociation between sensory loss and neuropathic pain and for developing diagnostic and therapeutic strategies that extend beyond conventional peripheral nerve assessment.

Therapeutic opportunities are of crucial importance. Oxidative stress emerges as a key mechanistic hub in diabetic neuropathy. A narrative review synthesised experimental and clinical evidence on oxidative stress related pathway, including hyperglycaemia – activated routes, mitochondrial dysfunction, endoplasmatic reticulum stress and chronic inflammatory signalling (4). New agents and emerging therapeutic targets for painful diabetic neuropathy were summarised by Panou et al. Promising outcomes have been reported for pilavapadin, an orally administered selective inhibitor of adapter protein-2-associated kinase 1 and vixotrigine, a broad spectrum voltage-gated sodium channel inhibitor. Experimental studies indicate a possible role for GLP-1RAs and SGLT2i in the treatment of neural damage. On the other hand, mainly small-size phase II shorter-term randomized controlled trials (RCTs) of different designs have been carried out, without clear evidence of positive effect of both GLP-1RAs and SGLT2i on clinical and neurophysiological neuropathic outcomes.

Ong et al. provide a comprehensive translational review of diabetic polyneuropathy, emphasizing distal sensory axon degeneration, failure of regenerative repair and the continuing lack of disease-modifying therapy. Their synthesis identifies pathways that may enhance axonal resilience and regrowth, while appropriately noting that many promising targets still require validation across experimental models and clinical trials. Complementing this axonal perspective, Wu et al. focus on Schwann-cell injury and the interplay of apoptosis, autophagy, pyroptosis, ferroptosis and necroptosis as potential therapeutic targets. Guo et al. review readily available laboratory parameters spanning metabolic, renal, hepatic, inflammatory, thyroid and vitamin-related markers and propose their integrated use for earlier detection and individualized risk stratification.

Several original studies address early detection and risk prediction. Current perception threshold testing showed moderate diagnostic accuracy for early C-fiber dysfunction in type 1 diabetes (Zhao et al.), whereas a random-forest model identified age, diabetes duration, HbA1c, glycated serum protein and 25-hydroxyvitamin D as major predictors of DPN (Li et al). Glycaemic variability was associated with the presence and severity of DPN independently of HbA1c in a propensity score-matched cohort, with systemic inflammation partly mediating this relationship (Yan and Wang). Mendelian randomization additionally suggested potentially protective associations of selected phosphatidylcholine species with diabetic neuropathy risk, opening a hypothesis-generating lipidomic perspective (Wang et al).

The therapeutic contributions span pharmacological, lifestyle and complication-oriented approaches. A meta-analysis of seven trials reported improved nerve conduction and glycaemic outcomes with dapagliflozin plus methylcobalamin, although the limited evidence base calls for larger, rigorously designed and more widely generalizable trials (Deng et al.). In a randomized study, both high-intensity interval training and moderate-intensity continuous training improved metabolic, inflammatory and neurological outcomes, with greater effects reported for high-intensity interval training (Guo et al.). Finally, a network meta-analysis of antimicrobial dressings for diabetic foot ulcers ranked antibiotic-impregnated bone cement and silver dressings most favorably, while underscoring the need for direct comparative and cost-effectiveness studies (He et al.).

Taken together, the contributions to this Research Topic illustrate the breadth of contemporary diabetic neuropathy research: from the Schwann cell and distal axon to the autonomic nervous system and brain, and from biomarkers and risk prediction to exercise, pharmacotherapy and management of foot complications. They also highlight the considerable heterogeneity of diabetic neuropathy regarding the nerve fibers involved, sensory manifestations, pain, underlying mechanisms and individual disease trajectories.

This heterogeneity argues against considering diabetic neuropathy as a single uniform entity and emphasizes the need for a more personalized approach to its diagnosis and treatment. Detailed phenotyping integrating clinical characteristics with quantitative sensory testing, neurophysiology, biomarkers and advanced imaging may help to identify clinically and mechanistically distinct patient subgroups. Longitudinal sensory profiling has demonstrated distinct trajectories of sensory dysfunction during the course of diabetic polyneuropathy (5). Importantly, sensory phenotypes may also reflect underlying structural nerve alterations: magnetic resonance neurography revealed distinct patterns of sciatic nerve structural integrity associated with different peripheral sensory phenotypes in individuals with type 2 diabetes (6). Together, these findings support the integration of structural and functional measures to characterize neuropathy beyond conventional diagnostic categories.

Such mechanistic phenotyping may be particularly important for painful diabetic neuropathy, where similar clinical symptoms can arise from different pathophysiological processes. Recent work combining quantitative sensory testing with C-fiber–oriented electrical stimulation demonstrated a striking dissociation between heat pain perception and nociceptor excitability: individuals with markedly impaired heat pain could retain substantial electrically evoked pain responses (7). This mismatch suggests that loss of a sensory function does not necessarily imply loss of the corresponding excitable axons and highlights the importance of distinguishing impaired sensory transduction from more advanced denervation. Such mechanistic differences may ultimately be relevant for patient stratification and the development of mechanism-based treatments.

Ultimately, advances in deep phenotyping, pain research, advanced imaging and mechanistic characterization should converge towards a precision-medicine approach in which patients are stratified according to their individual neuropathic phenotype and underlying pathophysiology. Linking these phenotypes and mechanisms to treatment response represents an important next step towards individualized therapeutic strategies (**Figure 1**). Thus, earlier and multidimensional phenotyping is essential, but mechanistic insights must now be translated into robust prospective studies, targeted therapeutic approaches and, ultimately, disease-modifying interventions.

**Figure 1:**
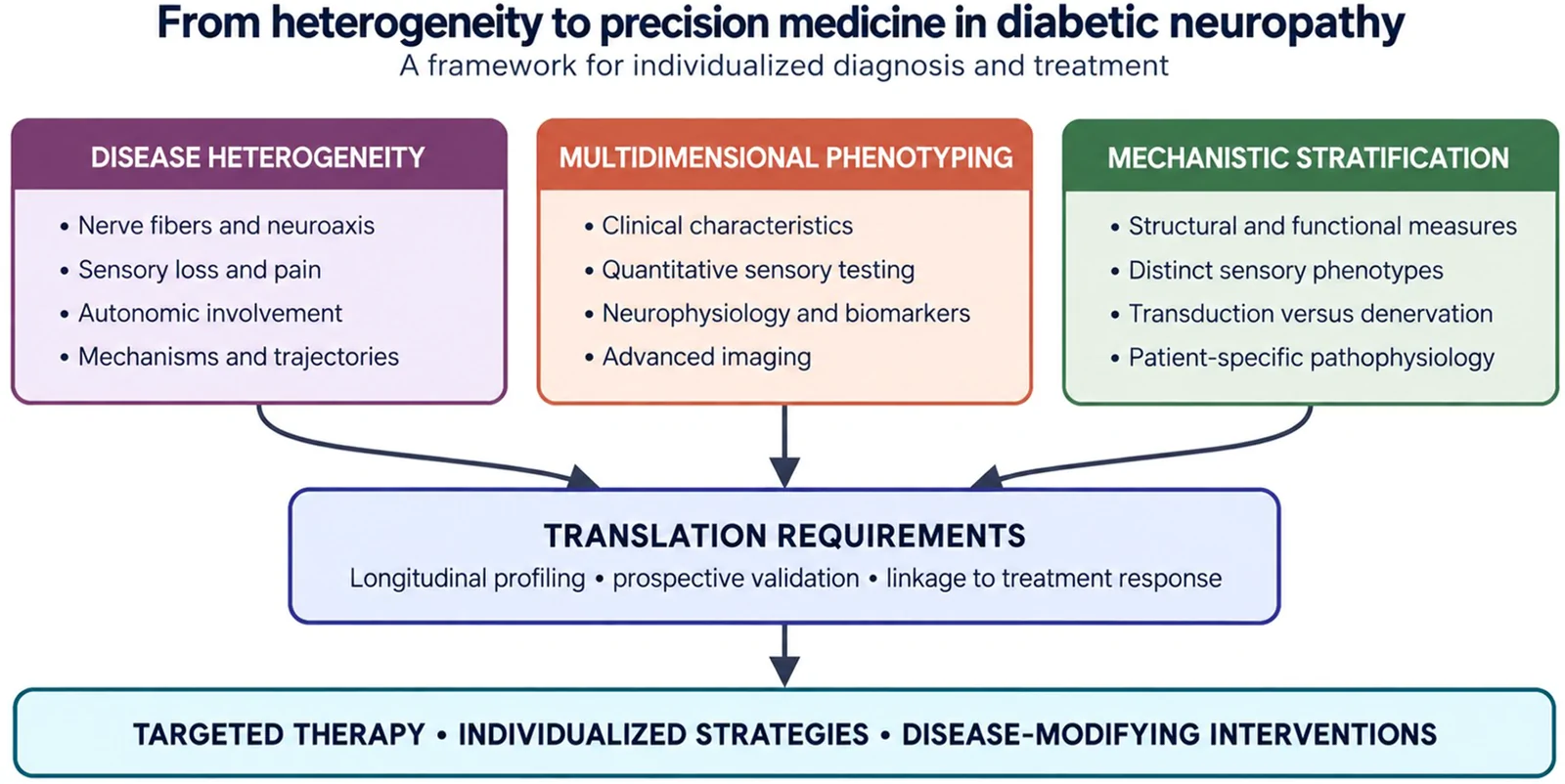
From heterogeneity to precision medicine in diabetic neuropathy. Multidimensional phenotyping integrating clinical characteristics, quantitative sensory testing, neurophysiology, biomarkers, and advanced imaging may enable mechanistic patient stratification. Longitudinal validation and linkage to treatment response are essential for developing individualized, mechanism-based, and ultimately disease-modifying interventions.
